# Management of rare intraventricular empyema secondary to late onset neonatal meningitis in an infant: A case report

**DOI:** 10.1016/j.ijscr.2025.111791

**Published:** 2025-08-12

**Authors:** Selemon Gebrezgabiher Asgedom, Dejen Tekiea Gebrewahd, Million Gebrewold Abdi, Yoseph Abebe Wondie, Ephrem Ashagrie Melese, Kefyalew Taye Belete

**Affiliations:** a School of Medicine, Yekatit 12 Hospital Medical College, Addis Ababa Health Office, Federal Ministry of Health, Ethiopia; b Department of Surgery, Neurosurgery Division, Addis Ababa University, Addis Ababa, Ethiopia; c ALERT Comprehensive Specialized Hospital, Federal Ministry of Health, Addis Ababa, Ethiopia; d Department of Diagnostic Radiology, Yekatit 12 Hospital Medical College, Addis Ababa Health Office, Federal Ministry of Health, Ethiopia; e Department of Public Health, College of Health Sciences and Referral Hospital, Ambo University, Ambo, Ethiopia

**Keywords:** Intraventricular empyema, Neonatal meningitis, Neuroendoscopic lavage, External ventricular drainage, Hydrocephalus, Ventriculitis

## Abstract

**Introduction and importance:**

Intraventricular empyema (IVE) is life-threatening intracranial infection characterized by the accumulation of purulent material within the brain's ventricular system. It is a rare complication of bacterial meningitis in neonates. *Escherichia coli*, a common cause of late-onset neonatal meningitis, can occasionally lead to severe complications such as ventriculitis and IVE. Early diagnosis and intervention are critical to preventing long-term neurological sequelae or death.

**Case presentation:**

A 44-day-old male Ethiopian infant presented with fever, irritability, vomiting, poor feeding, and abnormal body movements. Physical examination revealed bulging fontanelle and lethargy, suggesting raised intracranial pressure. Cerebrospinal fluid analysis confirmed bacterial meningitis, and brain imaging revealed bilateral intraventricular abscesses. The patient was diagnosed with intraventricular empyema secondary to late-onset meningitis caused by *Escherichia coli*. Management included intravenous administration of meropenem, external ventricular drainage to relieve pressure and remove purulent material, Neuroendoscopic lavage for direct intraventricular cleaning, and later placement of a ventriculoperitoneal shunt to manage hydrocephalus. The patient responded well to treatment, showing marked clinical improvement. The infant recovered well and demonstrated appropriate developmental progress at three-month follow-up.

**Clinical discussion:**

This case underscores the critical role of prompt diagnosis and a comprehensive therapeutic strategy in managing neonatal intraventricular empyema. Integration of neuroimaging, appropriate antimicrobial therapy, and endoscopic surgical interventions significantly contributed to the favorable outcome. Multidisciplinary care remains essential for addressing complications such as hydrocephalus and ensuring long-term recovery.

**Conclusion:**

Early recognition and integrated medical-surgical intervention can significantly improve outcomes in an infant with intraventricular empyema. Advanced neuroimaging, targeted antibiotics, and Neuroendoscopic techniques are pivotal in managing this life-threatening condition.

## Highlights

• Intraventricular empyema (IVE) is a rare but life-threatening complication of neonatal bacterial meningitis involving pus accumulation within the brain's ventricles
• A 44-day-old infant with *E. coli*-induced meningitis developed IVE, confirmed by MRI showing ventricular debris and ependymal enhancement.
• Treatment included intravenous and intraventricular antibiotics, Neuroendoscopic lavage, and later VP shunt for post-infectious hydrocephalus.
• Despite the severity, the infant made a full recovery with normal neurodevelopment at 3-month follow-up.

## 1 Introduction

Intraventricular empyema (IVE) is a life-threatening condition characterized by the accumulation of purulent material within the brain's ventricular system. It is an uncommon complication of intracranial infections, especially in neonates, with a global incidence estimated at less than 0.3 % among patients suffering from bacterial meningitis. [1]. It occurs less frequently than related infections such as subdural empyema or brain abscess [2,3]. In neonates, *Escherichia coli* is a leading cause of late-onset meningitis and is known for its ability to penetrate the blood-brain barrier and disseminate through cerebrospinal fluid, increasing the risk of ventriculitis and intraventricular empyema [4,5].

While neonatal meningitis and severe central nervous system infections remain a significant health burden in sub-Saharan Africa, including Ethiopia [6], there is a paucity of published data on the incidence, clinical features, and outcomes of IVE in this region. This lack of epidemiological information limits the development of targeted prevention and treatment strategies, underscoring the importance of case reports and studies that document regional experiences.

Diagnosis of IVE relies heavily on neuroimaging, with magnetic resonance imaging (MRI) providing superior sensitivity for detecting intraventricular pus and associated complications such as ventriculitis [5]. Management requires a multidisciplinary approach, combining empirical broad-spectrum antibiotics, surgical interventions like external ventricular drainage (EVD), and, in some cases, adjunctive intraventricular antibiotic therapy [7,8]. Recently, Neuroendoscopic ventricular irrigation has emerged as a promising minimally invasive technique that may improve outcomes by facilitating direct removal of purulent material [9,10].

This case report contributes to the limited literature by describing a rare case of *E. coli*-induced intraventricular empyema in a neonate successfully managed with Neuroendoscopic lavage. It highlights the critical importance of early diagnosis, multidisciplinary care, and the potential role of advanced Neuroendoscopic interventions, especially in resource-limited settings such as Ethiopia.

## 2 Case presentation

A 44-day-old male Ethiopian infant, born at term via spontaneous vaginal delivery to a 32-year-old para 5 mother, presented with a 3-week history of intermittent high-grade fever, history of poor breastfeeding, irritability, vomiting, and 3-day abnormal body movements occurring 2 to 3 times daily. The pregnancy was complicated by prolonged rupture of membranes (PROM) lasting over 24 h.

Initially, the infant was admitted to a regional general hospital where he was diagnosed with late-onset neonatal sepsis with central nervous system involvement. He was treated empirically with intravenous ampicillin at 50 mg/kg every 8 h and gentamicin at 5 mg/kg once daily, along with supportive care including suppository paracetamol at 10–15 mg/kg every 6 h for fever, and phenobarbital at 5 mg/kg loading dose followed by 3 mg/kg/day in divided doses for seizure control. Despite completing a 3-week course of therapy at the regional hospital, the infant showed no clinical improvement. The family resides in a rural area with limited access to specialized healthcare and low health literacy, which contributed to delayed referral.

Due to persistent high-grade fever, worsening seizures, and progressive altered mental status, he was referred to our tertiary hospital for further evaluation and management. On admission, physical examination revealed a febrile infant with a temperature of 39.3 °C, lethargic that responds to voice, bulging anterior fontanelle, generalized hypertonia, and absence of Moro reflex. Pupillary reflexes were sluggish but equal bilaterally, and no focal neurological deficits were noted. Signs of increased intracranial pressure, including vomiting and irritability, were present. The infant appeared moderately dehydrated with dry mucous membranes but no petechiae or skin rashes.

On admission Laboratory investigations showed leukocytosis (21,500/mm^3^) with 71 % neutrophils and an elevated C-reactive protein (127 mg/L). Cerebrospinal fluid (CSF) analysis revealed marked pleocytosis (1776 cells/mm^3^, 86 % neutrophils), high protein (593 mg/dL), and hypoglycorrhachia (glucose 5 mg/dL). Blood and CSF cultures isolated *Escherichia coli* sensitive to gentamicin, amikacin, meropenem, ciprofloxacin, and chloramphenicol.

**Fig. 1:**
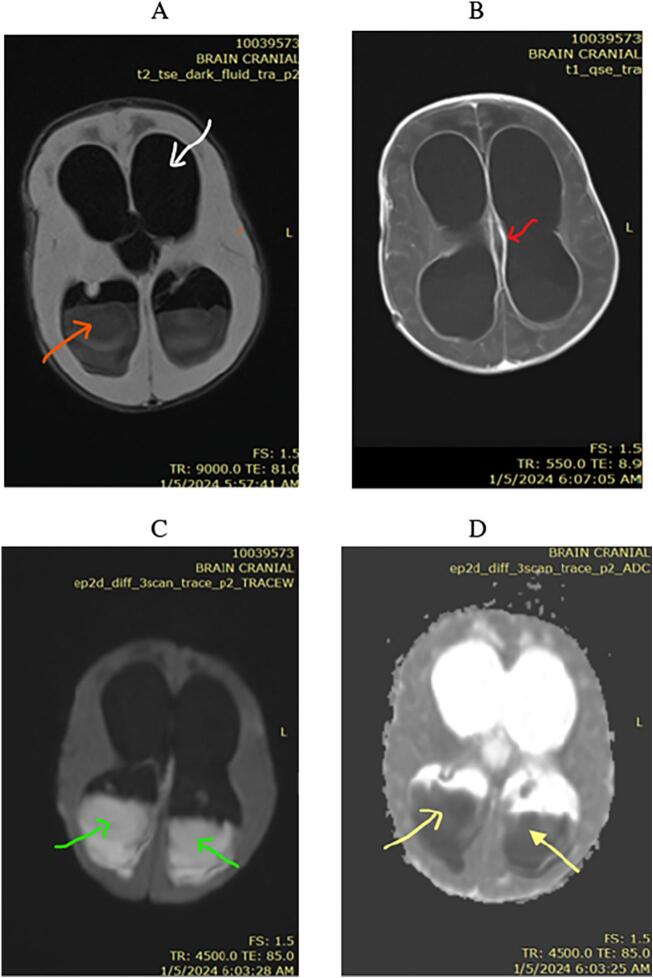
a. FLAIR ventriculomegaly (white arrow) with hyperintense intraventricular material (red arrow). b. Post-contrast T1-weighted sequences reveal Enhancement of the ventricular walls (red arrow). c. DWI- demonstrates marked hyperintensity (green arrows). d. ADC; Low apparent diffusion coefficient (yellow arrows).

Cranial ultrasound via the anterior fontanelle demonstrated irregular, echogenic collections within the bilateral lateral ventricles, involving the posterior and temporal horns. The ventricular walls were thickened and hyperechoic, indicating ventriculitis and intraventricular abscess formation. Given limited availability, diagnostic accuracy and to avoid radiation exposure in the neonate, magnetic resonance imaging (MRI) was prioritized over CT. Brain MRI findings include ventriculomegaly with hyperintense intraventricular material on FLAIR imaging (Fig. 1a). Post-contrast T1-weighted sequences (Fig. 1b) reveal enhancement of the ventricular walls, consistent with ependymal inflammation (ependymal enhancement) indicative of ventriculitis. Diffusion-weighted imaging (DWI) (Fig. 1c) demonstrates marked hyperintensity within the intraventricular abscess, with corresponding low apparent diffusion coefficient (ADC) values (Fig. 1d), confirming restricted diffusion characteristic of a purulent intraventricular collection (empyema). Clinical Timeline and follow up is presented in Table 1.

**Table 1:** Clinical timeline and follow up until age of 90 days.

Age (days)	Clinical findings	Investigations	Treatment/interventions	Comments
0	Born at term, spontaneous vaginal delivery	–	Routine neonatal care	Pregnancy complicated by PROM (>24 h)
21 and 22	Onset of intermittent high-grade fever, poor breastfeeding, irritability and vomiting.	CBC, Lumbar puncture (LP) done for CSF analysis (cell count, glucose and protein) at regional hospital Investigation result CBC- WBC-17,122(84 % neutrophils) CSF analysis: pleocytosis 143 cells/mm^3^, 79 % neutrophils, protein 278 mg/dL, glucose 25 mg/dL	Admission to regional hospital Empirical IV ampicillin (50 mg/kg q8h) and gentamicin (5 mg/kg daily); suppository paracetamol (10–15 mg/kg q6h)	CSF culture and CRP were not sent do to unavailability Diagnosed with late-onset neonatal sepsis with CNS involvement
22–42	Persistent high-grade fever; new onset of seizures at day 39; no clinical improvement	Periodic labs (CBC was updated 3 times ranging from 16,430 to 22, 856 with neutrophilic predominance and gradual increment)	Continued initial antibiotics, phenobarbital (5 mg/kg loading, then 3 mg/kg/day divided) and supportive care	Treatment failure suspected at the end of 3 weeks treatment
43	Referral to tertiary hospital for further management	Admission labs: WBC 21,500/mm^3^ (71 % neutrophils), CRP 127 mg/L	Transferred by Ambulance	Patient received at Zewditu memorial hospital
44	Physical exam: fever 39.3 °C, lethargy, bulging fontanelle, hypertonia, absent Moro reflex, vomiting, irritability	CSF analysis: pleocytosis (1776 cells/mm^3^, 86 % neutrophils), protein 593 mg/dL, glucose 5 mg/dL; Blood & CSF cultures positive for *E. coli* sensitive to gentamicin, amikacin, meropenem, ciprofloxacin, chloramphenicol; Cranial ultrasound: echogenic intraventricular collections; ventricular wall thickening	Meropenem initiated (20 mg/kg q8h)	Imaging and labs confirmed intraventricular empyema
46	Persistent fever and worsening symptoms; decision for surgery	MRI: ventriculomegaly, ventricular wall enhancement, intraventricular abscess with diffusion restriction	Under general anesthesia: Neuroendoscopic ventricular lavage and septostomy via Keen's point; EVD placed via Kocher's point; ventricles irrigated with Ringer's lactate; intrathecal gentamicin 2 mg administered intraoperatively	Informed consent obtained; nasogastric tube used as ventricular catheter
47–49	Clinical improvement: breastfeeding resumed on Day 48; seizures controlled	Serial US: improved ventricular echogenicity; serial CBC & CSF every 48 h.	Continued IV meropenem; intrathecal gentamicin 2 mg daily for 5 days via EVD	Favorable clinical and lab response noted early
50–69	Continued improvement; EVD maintained	CSF WBC counts declining; CRP trending down	Continued IV meropenem; monitoring of shunt indication	Residual hydrocephalus detected on serial imaging
70	No new neurological deficits; persistent hydrocephalus	Repeat CSF: WBC 24 cells/mm^3^ (64 % lymphocytes), protein 123 mg/dL, glucose 56 mg/dL	Medium-pressure ventriculoperitoneal (VP) shunt inserted	No complications during surgery; shunt functional
71–90	Continued recovery; discharged on oral antibiotics	Discharge labs: stable	Plan for follow-up at 2 weeks, 1 month, and 3 months; neurodevelopmental screening initiated	Denver II assessment at 3 months showed age-appropriate milestones

## 3 Management

On admission, the infant was initially stabilized with bolus intravenous fluids. Cerebrospinal fluid (CSF) analysis and brain MRI were promptly performed, confirming bacterial meningitis complicated by intraventricular empyema. Blood and CSF cultures subsequently isolated *Escherichia coli* after 48 h. Empirical intravenous antibiotics ampicillin and gentamicin had been initiated at the regional hospital and continued admission. However, given the lack of clinical improvement over three weeks and culture sensitivities confirming *E. coli*, antibiotic therapy was escalated to intravenous meropenem at 40 mg/kg every 8 h.

On the same day, after thorough discussion with the parents regarding the infant's condition and the need for Neuroendoscopic surgery with ventricular irrigation, written informed consent was obtained. Under general anesthesia, the infant underwent Neuroendoscopic surgical intervention. An external ventricular drain (EVD) was inserted through a right Kocher's point burr hole using a size 10 nasogastric tube as the catheter. Through a separate right Keen's point burr hole, Neuroendoscopic ventricular lavage and septostomy were performed. The ventricles were irrigated with warmed Ringer's lactate solution. Intraoperatively, intrathecal gentamicin at 2 mg was administered via the EVD, and this was continued postoperatively for an additional five consecutive days.

Following the procedure, there was a gradual decrease in fever and resolution of abnormal body movements, which became fully controlled with ongoing anticonvulsant therapy. By the second postoperative day, the infant began breastfeeding effectively, indicating early clinical recovery. Serial transfontanel ultrasound imaging, repeated CSF analyses every 48 h, and laboratory monitoring including complete blood count (CBC) and C-reactive protein (CRP) were performed regularly to assess treatment response. At the end of the fourth week, repeat CSF analysis showed marked improvement, with a white blood cell count of 24 cells/mm^3^ (64 % lymphocytes), protein level of 123 mg/dL, and glucose of 56 mg/dL findings consistent with resolving infection.

The EVD was maintained for two weeks alongside intravenous meropenem. The patient continued to improve; however, persistent communicating hydrocephalus was detected on follow-up imaging. On the 4th week, a medium pressure ventriculoperitoneal (VP) shunt was inserted to manage the hydrocephalus without intraoperative complications. The shunt remained functional throughout hospitalization, with no signs of blockage or infection. The infant demonstrated marked clinical improvement and seizure control following these interventions. After 28 days of hospitalization at the tertiary center, he was discharged on oral antibiotics with plans for close outpatient follow-up.

Post-discharge, the infant was followed regularly at neurology and neurosurgery clinics, with visits at 2 weeks, 1 month, and 3 months post-discharge. Neurodevelopmental assessments using Denver II screening indicated age-appropriate progress at 3 months. The VP shunt remained functional, with no signs of malfunction or infection.

Seizure control was maintained with tapering doses of phenobarbital. No adverse drug reactions or surgical complications such as EVD blockage or shunt failure were reported during the follow-up period.

Initially, the infant's parents were understandably anxious about their child's condition. Through clear communication, detailed counseling, and comprehensive multidisciplinary care, they developed confidence in the treatment process and expressed gratitude following their child's successful recovery.

## 4 Discussion

Intraventricular empyema (IVE) is life-threatening complication of neonatal bacterial meningitis, often resulting in poor neurological outcomes and high mortality [1,5]. This report describes a case of *Escherichia coli*-induced IVE following late-onset neonatal meningitis in Ethiopia. The infant presented with persistent fever, seizures, and signs of increased intracranial pressure after ineffective empirical therapy at a regional hospital. This case illustrates the importance of early diagnosis, access to advanced imaging, and multidisciplinary care including neurosurgical intervention in improving outcomes.

IVE occurs less frequently than other intracranial infections such as subdural empyema or brain abscess [7]. While *E. coli* is a common cause of late-onset neonatal meningitis, it more rarely leads to IVE, especially in term infants without underlying anatomical abnormalities [6,8]. The infant in this case had risk factors including prolonged rupture of membranes, which increases susceptibility to gram-negative sepsis and CNS invasion [11]. The clinical presentation fever, irritability, bulging fontanelle, and abnormal movements were typical of ventriculitis and IVE, as reported in other pediatric cohorts [12]. Initial cranial ultrasound suggested intraventricular pus, but MRI provided definitive findings: ependymal enhancement, hyperintense ventricular contents, and restricted diffusion hallmarks of empyema [2,5]. While MRI offers superior sensitivity, access is limited in many Ethiopian settings, often delaying appropriate surgical intervention. Prioritization of MRI in this case contributed to the favorable outcome, despite resource constraints.

Treatment combined intravenous meropenem chosen for its excellent CSF penetration and gram-negative coverage [3] with intraventricular gentamicin via an external ventricular drain (EVD). This multimodal approach aligns with current recommendations and evidence supporting intraventricular antibiotics in severe ventriculitis [7,8]. Clinical improvement, including control of seizures and resumption of breastfeeding, began within two days of post-intervention. Neuroendoscopic ventricular lavage and septostomy were performed to directly evacuate purulent material. This technique, though not widely available in sub-Saharan Africa, has been associated with improved infection control and reduced need for multiple interventions in pediatric patients [10,12]. While no intraoperative complications occurred, the child developed post-infectious communicating hydrocephalus, a common sequela of IVE [12,13] necessitating VP shunt placement in the fourth week. Even though there is evidence supporting the superiority of Endoscopic Third Ventriculostomy (ETV) over ventriculoperitoneal shunting (VPS) for post-infectious hydrocephalus [14], the ETV Success Score of Kulkarni for this patient was 20, a low score considering the infant's age (below 6 months), post-infectious etiology, and absence of previous shunt history [15], making this patient a poor candidate for ETV.

At three-month follow-up, the infant had age-appropriate development (Denver II screening) and no shunt complications, underscoring the potential benefit of early and aggressive treatment. However, given the rarity of reported IVE cases in Ethiopia or broader sub-Saharan Africa, generalizability remains limited. There is a need for regional surveillance data to better understand incidence, outcomes, and access disparities related to advanced neurocritical care. While this case shows promising outcomes, conclusions must be tempered by the single-patient context. Nevertheless, it adds to a growing body of literature supporting early neuroimaging, targeted antimicrobial therapy, and Neuroendoscopic lavage in managing neonatal IVE. Future studies should evaluate cost-effectiveness and long-term neurodevelopmental outcomes in resource-limited environments.

## 5 Limitations

This case report describes a single patient, limiting the ability to generalize findings across broader neonatal populations. Due to resource constraints in Ethiopia, several diagnostic and therapeutic limitations were encountered. A standard external ventricular drainage (EVD) system was not available, necessitating the use of a nasogastric (NG) tube as a substitute, a common but suboptimal practice in low-resource neurosurgical settings. Additionally, postoperative MRI was not performed due to limited access. Furthermore, delayed referral and absence of early cultures at the regional hospital contributed to disease progression before transfer to a tertiary center.

## 6 Conclusion

This case report highlights the rare occurrence of intraventricular empyema secondary to late-onset neonatal meningitis, emphasizing the importance of early diagnosis and a multidisciplinary approach. Timely intervention with antibiotics, Neuroendoscopic lavage, and ventricular drainage significantly improved the patient's clinical outcome, underscoring the role of combined medical and surgical management in such life-threatening infections.

## Abbreviations

CSF: Cerebrospinal Fluid
EVD: External Ventricular Drainage
FLAIR: Fluid-Attenuated Inversion Recovery sequence
MRI: Magnetic Resonance Imaging
DWI: Diffusion Weighted Imaging
ADC: Apparent Diffusion Coefficient
VPS: Ventriculoperitoneal shunt
PROM: Prolonged Rupture of Membranes

## Consent for publication

Written informed consent was obtained from the patient's legal guardians for the publication of this case report, including accompanying images and clinical details. All ethical principles outlined in the Declaration of Helsinki were adhered to, and patient confidentiality was strictly maintained.

## Ethics approval and consent to participate

Not applicable.

## Guarantor

Selemon Gebrezgabiher Asgedom

## Research registration number

This case report is not registered; it is not a clinical Trial.

## Funding

The authors received no financial support for the research, authorship, and/or publication of this article.

## Author contribution

Conceptualization: Selemon Gebrezgabiher Asgedom. Case narration: Selemon Gebrezgabiher Asgedom, Yoseph Abebe Wondie, Kefyalew Taye Belete. Manuscript review and editing: Dejen Tekiea Gebrewahd, Million Gebrewold Abdi, Yoseph Abebe Wondie, Ephrem Ashagrie Melese.

## Declarations

The case report has been reported in line with the SCARE criteria [16].

## Conflict of interest statement

The authors declare that they have no competing interests.

## Data Availability

The data supporting the findings of this case report are available within the article. Additional information is available from the corresponding author upon reasonable request.
